# Life satisfaction and resilience in medical school – a six-year longitudinal, nationwide and comparative study

**DOI:** 10.1186/1472-6920-6-48

**Published:** 2006-09-19

**Authors:** Kari Kjeldstadli, Reidar Tyssen, Arnstein Finset, Erlend Hem, Tore Gude, Nina T Gronvold, Oivind Ekeberg, Per Vaglum

**Affiliations:** 1 Department of Behavioural Sciences in Medicine, Institute of Basic Medical Sciences, Faculty of Medicine, University of Oslo, Norway

## Abstract

**Background:**

This study examined the relationship between life satisfaction among medical students and a basic model of personality, stress and coping. Previous studies have shown relatively high levels of distress, such as symptoms of depression and suicidal thoughts in medical undergraduates. However despite the increased focus on positive psychological health and well-being during the past decades, only a few studies have focused on life satisfaction and coping in medical students. This is the first longitudinal study which has identified predictors of sustained high levels of life satisfaction among medical students.

**Methods:**

This longitudinal, nationwide questionnaire study examined the course of life satisfaction during medical school, compared the level of satisfaction of medical students with that of other university students, and identified resilience factors. T-tests were used to compare means of life satisfaction between and within the population groups. K-means cluster analyses were applied to identify subgroups among the medical students. Analysis of Variance (ANOVA) and logistic regression analyses were used to compare the subgroups.

**Results:**

Life satisfaction decreased during medical school. Medical students were as satisfied as other students in the first year of study, but reported less satisfaction in their graduation year. Medical students who sustained high levels of life satisfaction perceived medical school as interfering less with their social and personal life, and were less likely to use emotion focused coping, such as wishful thinking, than their peers.

**Conclusion:**

Medical schools should encourage students to spend adequate time on their social and personal lives and emphasise the importance of health-promoting coping strategies.

## Background

To be a student in medical school may be stressful [1-3]. Previous studies have shown relatively high levels of distress, such as symptoms of depression [4,5] and suicidal thoughts [6,7] in medical undergraduates. Less is known about what conditions encourage positive mental health, and a recent review of research on medical student distress emphasised the need for research concerning the factors that promote well-being [8]. Despite increased attention being paid to positive psychological health and well-being during the past decades [9,10], only a few studies have focused on life satisfaction and coping in medical students. Of these, one study found that problem focused and emotion focused coping related positively to physical health in first year medical students [11], and another study found that coping strategies characterised by engagement predicted fewer symptoms of depression compared to disengagement strategies [12]. A qualitative study of medical student perceptions of an elective wellness course reported positive responses from the students [13]. A recent study concluded that personal statements and referees' reports used in medical school applications can not predict who will be satisfied or dissatisfied with a medical career [14]. To date, no longitudinal study has identified predictors of sustained high levels of life satisfaction among medical students.

The main purpose of this study was to examine the relationship between life satisfaction among medical students and a basic model of personality, stress and coping [15,16]. Personality traits, such as neuroticism and extroversion, have been found to be predictors of life satisfaction [17]. As indicated above, research on the psychological adaptation of medical students has focused on distress. Perceived medical school stress has been linked to current mental distress [18] and to forthcoming mental health problems [19], and is therefore assumed to affect life satisfaction. According to the resilience model, the way that students cope with stress factors may influence their mental health in medical school [15,20]. We anticipated that active or problem focused coping and seeking social support would promote higher satisfaction with life, whereas passive or emotion focused coping would have a negative impact on life satisfaction. In particular, we wanted to study the characteristics of students who sustained high levels of life satisfaction during medical school, and compare them with the characteristics of their peers to find factors of resilience that may be used to make positive changes, and hence help future medical students [21]. We assumed that stress factors pertaining to medical school might have a negative impact on students' life satisfaction, and wanted to examine if this effect can be counteracted with efficient coping strategies. We also anticipated that particular personality traits would affect students' susceptibility to the educational challenges.

A recent study from our group [22] followed a cohort of Norwegian physicians from their final term in medical school to the ninth postgraduate year and found lower levels of life satisfaction in the fourth and ninth postgraduate years compared to comparable population samples. The study also found that the level of life satisfaction was even lower at the end of medical school, but it did not include data from the undergraduate years. There are a number of studies which have compared levels of distress among medical students with that of other students [23-25], but to our knowledge there has been no comparative study focussed on positive mental health.

Against this background we have analysed data from a longitudinal, nationwide study of Norwegian students entering medical school in 1993 (n = 421) and compared them with two samples of non medical students. The medical students were assessed three times: at medical school entry, at mid curriculum (third year) and in the graduating semester (sixth year). The objectives of this study were: (I) to describe the course of life satisfaction in medical school students; (II) to compare the level of life satisfaction among medical students with that of other university students; and (III) to identify resilient medical students and assess whether these students differ from their peers in regards to personality, stress and coping.

## Methods

### Participants

All students entering medical school at the four Norwegian universities in 1993 were invited to participate. Data were collected by mail in their first, third, and final (sixth) year and were anonymous. Out of the initial pool of 421 students, 375 responded to the baseline assessment (89% response rate, 54% women, mean age 22.1 years (SD = 3.1)), 302 participated in the second assessment (72% response rate, 59% women, mean age 24.8 years (SD = 2.6)) and 287 took part in the final year assessment (68% response rate, 55% women, mean age 27.7 years (SD = 2.4)). A total of 236 students responded on all three occasions (56% of the original cohort, 60% women, mean age at baseline 21.6 years (SD = 2.5)), constituting the longitudinal sample to be investigated. We used the longitudinal sample in order to analyse the course of life satisfaction throughout medical school, but used all responses from the first and last assessments to compare the level of life satisfaction of medical students with that of other university students. The study was conducted according to the guidelines and with the approval of the regional ethical committees and the National Data Inspectorate in Norway.

The two samples used for comparison were obtained from a nationwide, cross-sectional study on quality of life from 1996 [26]. The inclusion criteria encompassed all university students in corresponding age groups. Among the 3500 participants there were 643 university students. 65 of these students (63% women, mean age 23.2 years (SD = 1.6)) matched the age of the medical students in their first year, while 136 students (51% women, mean age 27.7 years (SD = 1.9)) could serve as an age based comparison group for the medical students in their final year. We were unable to define a comparison sample based on age for the level of life satisfaction among the medical students in their third year that did not overlap with the comparison groups being used for the other two points of time.

### Materials and procedure

#### Life satisfaction

Life satisfaction was assessed on a 7-point Likert scale using a global one-item measure ("When you think about your life today, are you by and large very satisfied or very dissatisfied?") with alternatives ranging from (1) very dissatisfied, to (7) very satisfied. This item has been used in several other studies, such as the Canadian Community Health Survey [27] and the Nord-Trøndelag Health Survey [28]. The correlation between this item and a validated subjective well-being scale [29] is .72.

#### Subgroups

In order to identify subgroups based on the course of life satisfaction in medical school, we applied cluster analysis to the reported levels of life satisfaction in the longitudinal sample.

#### Personality

Personality was measured at baseline, using three out of four traits from the 36-item version of the Basic Character Inventory (BCI), a questionnaire constructed by Lazare, Klerman and Armor [30] and modified by Torgersen [31]. Each of the trait dimensions is based on nine questions with a dichotomous response choice (agree/do not agree), thereby comprising a total score ranging from 0 (low) to 9 (high). Together they correspond to the classic "big three" personality traits. The BCI – vulnerability scale (Cronbach's alpha = .68) measures the neuroticism dimension, the BCI – intensity scale (Cronbach's alpha = .68) assesses extroversion/introversion, and the BCI – control scale (Cronbach's alpha = .67) describes the degree of compulsiveness/obsessiveness.

#### Medical school stress – academic worries and social and personal renunciations

Stress factors linked to medical school were measured in the middle and final years, using eight items from the 13-item Perceived Medical School Stress (PMSS) instrument, developed by Vitaliano et al. [18]. The PMSS items use a 5-point Likert scale ranging from (1) I strongly disagree, to (5) I strongly agree. A principal component analysis with varimax rotation of these eight items yielded a scree plot with a two-factor solution of six items. The first factor, PMSS – academic worries (Cronbach's alpha = .81), consists of three items that address concerns about mastering medical knowledge and ability to endure the long hours and responsibilities. The second factor, PMSS – social and personal renunciations (Cronbach's alpha = .70), consists of three items that assess the extent to which the student feels that medical school controls one's life and educates physicians at the expense of one's personality and interests. In accordance with the longitudinal outcome variable and in order to identify perceived stress over an extended period of time, each of the two factors is measured by the sum of the scores of its respective items in the last two assessments (scale: 6–30).

#### Coping

The coping strategies of the medical students were measured in their middle year, using the 42-item Ways of Coping Checklist (WCCL) [20], with a 5-point Likert scale ranging from (0) does not describe me at all, to (4) describes me in most situations. This instrument was originally divided into five coping dimensions (problem focused, self-blaming, seeking social support, wishful thinking and avoidance), but when we performed a principal component analysis (varimax rotation), the scree plot indicated a three-factor solution with 26 items. The first component, WCCL – problem focused (Cronbach's alpha = .75, scale: 0–40), is comprised of ten items concerning problem focused coping; the second, WCCL – social support (Cronbach's alpha = .77, scale: 0–20), consists of three items about seeking social support and two items about not avoiding stress factors; and the third component, WCCL – wishful thinking (Cronbach's alpha = .80, scale: 0–44), is made up of eight questions dealing with wishful thinking, two questions about self-blaming and one question regarding avoidance.

Table 1 shows the distribution of the explanatory variables in the longitudinal sample.

#### Statistics

We used t-tests to compare means of life satisfaction between and within the population groups. In order to identify the subgroups we applied K-means cluster analyses. To compare the subgroups, we made use of Analysis of Variance (ANOVA) and logistic regression analyses.

## Results

### Course of life satisfaction in medical school (longitudinal sample)

The mean values of life satisfaction among the medical students in the longitudinal sample were 5.7 (SD = 1.0) in the first year, 5.3 (SD = 1.1) in the third year, and 5.3 (SD = 1.2) in the final year. A paired samples t-test showed there was a statistically significant reduction in life satisfaction from baseline to the middle year (t (df) = 5.2 (235), p < .01), but no statistically significant change during the last three years. This applied to both genders. The 139 students who responded to the first assessment, but then dropped out and did not respond to the second and third assessments, reported a lower level of life satisfaction (mean = 5.4, SD = 1.1) when entering medical school than those who constituted the longitudinal sample (mean = 5.7, SD = 1.0). An independent samples t-test showed this difference to be significant (t (df) = -2.3 (373), p = .02).

### Comparison with other students (cross-sectional samples)

On entering medical school, the level of life satisfaction among the medical students in the cross-sectional sample (mean = 5.6, SD = 1.0) was not significantly different from that of other university students of the same age (mean = 5.4, SD = .9) (t (df) = 1.0 (438), p = .32). In their final year, however, the level of life satisfaction among the medical students (mean = 5.3, SD = 1.2) was significantly lower than among other students in the same age group (mean = 5.6, SD = 1.0) (t (df) = -2.8 (327.7), p = .01). This applied to both genders.

### Characteristics of the two subgroups (longitudinal sample)

First, we ran an analysis with three clusters and found one group with increasing life satisfaction (n = 44), one with decreasing life satisfaction (n = 67) and one group with stable high life satisfaction (n = 125). After performing analysis of variance (ANOVA) of the explanatory variables and the three clusters, it became evident that the stable life satisfaction group differed from the changing life satisfaction groups. These fluctuating groups showed many similarities regarding personality, perception of medical school stress and coping strategies. At first we considered the students with increasing life satisfaction as being adaptive and expected them to stand out when compared with other students. However, the similarities between the students with increasing and decreasing life satisfaction, and the differences between these and the ones with stable high life satisfaction, forced us to interpret these findings as indicating that there were really only two true subgroups; one with sustained high life satisfaction, and one with fluctuating levels. We therefore ran a second analysis with two clusters, and found a stable group (n = 149) and a group with fluctuating levels of life satisfaction (n = 87). Since our main interest was describing the resilient students, we decided to employ the two-cluster solution, with one stable and one labile group, and applied logistic regression analysis to identify predictors of stability versus fluctuation. Table 2 shows the levels of life satisfaction in medical school for the two subgroups.

The two groups were compared in a personality, stress and coping model in order to see if they differed in the personality traits vulnerability, intensity and control (BCI), whether they had different levels of academic worries or perceived medical school as interfering more or less with their social and personal life (PMSS), and what type of coping strategies they used, such as being problem focused, seeking social support or focusing on emotions like wishful thinking (WCCL). The crude and adjusted associations between the explanatory variables and the two subgroups are shown in table 3. Unadjusted analyses showed that the students with stable high life satisfaction scored lower on the personality trait vulnerability, had less academic worries, perceived medical school as interfering less with their social and personal life, were more likely to cope with stress by using a problem focused approach and seeking social support and were less likely to turn to wishful thinking. In the adjusted model, perception of social and personal renunciations (Odds Ratio (OR) = .76 [.68–.85], p < .01) and wishful thinking (OR = .93 [.87–.1.00], p = .04) were the only explanatory variables which maintained statistical significance. Additional analyses showed that the statistical effect which the personality trait vulnerability had on the course of life satisfaction seemed to be redirected through the coping strategy wishful thinking in the adjusted model.

## Discussion

The level of life satisfaction among the medical students decreased from their first to their third year in medical school, and remained at this lower level until graduation. The comparison analyses showed that the medical students were as satisfied as other students when starting their studies of medicine, but reported a lower level than the comparison group in their final year. Furthermore, stable high levels of life satisfaction in medical school corresponded to low levels of stress in the form of one's perception of social and personal renunciations, and by low levels of wishful thinking as a way of coping.

How do we explain the decrease in life satisfaction during the first years in medical school? We do not know the level of life satisfaction among the students before they embarked on their studies of medicine. It is possible that they reported higher levels of satisfaction than they normally would due to the fact that they had recently been accepted to medical school, which has Norway's highest admission requirements and that they simply returned to their normal level of satisfaction by the third year in medical school. However, the fact that medical students were as satisfied as other students when entering medical school, but less satisfied at graduation, contradicts this explanation, and indicates that the reduction in life satisfaction may have been caused by factors specific to medical school. It may be that the medical students did not adapt to their studies as well as other students. Although some studies have found that pharmacy students [23], graduate science students, [24,25] and law students [32] experience even more distress than medical students, the presumption that medical school has an impact on medical students' mental health is supported by several studies, which have shown that the medical students' well-being deteriorates during medical school. A longitudinal study of depression among medical students found that upon entering medical school the students' emotional status resembled that of the general population, but that depression scores rose and remained high during medical school [33]. This is consistent with findings of lower levels of life satisfaction among Canadian medical residents than among the general population [27]. Similar results have also been found in our group when comparing Norwegian medical graduates and physicians to comparable population samples in Norway [22]. Although the mean levels of life satisfaction among the medical students were quite high throughout medical school, our study supports the notion of the medical education and career as having an unfavourable effect on general life satisfaction among students and physicians. According to two review articles by Dyrbye et al, the impact of this effect has not been thoroughly studied, but some results indicate that student distress may correlate with impaired academic performance, cynicism, academic dishonesty, substance abuse and suicide [8,34].

The main aim of this study was to identify resilient medical students and find out if these students differed from their peers in personality, perceived stress, and coping strategies. We succeeded in finding a subgroup of students with stable high levels of life satisfaction. Compared to the group with fluctuating levels of life satisfaction, the stable students differed with respect to both perceived stress and coping abilities.

Our study found that low scores on the PMSS – social and personal renunciations item predicted stable high life satisfaction, even when susceptible personality traits were controlled for. This indicates that students in the stable group perceived medical school as interfering far less with their social and personal life than did students with fluctuating levels of life satisfaction. The ability to find enough energy to spend on several of life's domains, while maintaining a balance between them may be crucial for experiencing stable high life satisfaction. This finding is supported by previous studies which have shown that socializing decreased in medical school [35], that inadequate social activity was linked to impaired psychological health among medical students [36], and that leisure activities can reduce stress in medical school [37]. Another study revealed that many medical students felt guilty for spending time on social activities and personal well-being, although they recognised the importance of doing so [13]. Medical school should encourage students to maintain their outside interests and leisure activities and to make time for friends and recreation.

In the univariate analysis, low levels of academic worries corresponded to stable high life satisfaction. Although this effect did not remain statistically significant in the adjusted model, this finding may still indicate that perceived cognitive abilities are important for life satisfaction among medical students.

As expected, we found that a low level of passive, emotion focused coping, such as wishful thinking, was associated with stable high life satisfaction. Other studies support the harmful effect of emotion focused coping among medical students [12,38]. Studies of another cohort of Norwegian physicians showed that this form of coping during medical school predicts forthcoming mental health problems [19]. Although some of these studies found active coping to promote well-being [11] and to protect against depression [12], these factors reached predictive power in the unadjusted analyses of our study, but not in the adjusted model. Coping strategies can be modified by educational and therapeutic interventions. Training in how to use healthy ways of coping should therefore be provided in medical school, for instance, through the implementation of stress management courses.

We found that personality traits did not predict stable high life satisfaction when we controlled for other coping variables. A low level of vulnerability predicted stable high levels of life satisfaction in the univariate analysis, but this effect seemed to be channelled through wishful thinking in the adjusted model. The role of personality and dispositional variables has been emphasised in several other life satisfaction studies [10,17]. However, another study from our research group found only a rather modest effect of personality on life satisfaction among medical postgraduates and physicians [22]. It seems that for people in this educational and occupational group, stress and coping may be more important for life satisfaction than personality.

While other studies have found that female medical students report more stress [4] and less satisfaction with life than male medical students [39], we found no gender differences in life satisfaction in our study. This is in accordance with earlier reported findings on mental health among Norwegian physicians [19,40] and may reflect the relatively equal position of the genders in Norway.

The major strength of this study is that it is a longitudinal study, following a nationwide cohort of medical students throughout their entire study period. Medical education in Norway consists of six years of education at the university, followed by one and a half years of compulsory residency. Although this is different from, for example the United States, our findings may be generalised beyond Norway, at least within the European system of training. High levels of stress are reported in several countries [1,3,4,12,36,37]. The response rates in the cross-sectional samples of our study were quite high, and although the response rate was somewhat lower in the longitudinal sample, it still consisted of more than half of all the students who entered medical school in Norway in 1993. Considering that those students who dropped out of the longitudinal sample after having responded the first year reported a lower level of life satisfaction when entering medical school than those who participated in all three assessments, the reduction in life satisfaction might have been even larger if all students had been part of the longitudinal sample (type II error). The data on the control groups were collected midway through the study. The comparisons made between the students in their first and last years are hence done with data of other students collected three years later and three years earlier, respectively. We consider any possible effect of this difference in time to be minimal. Another limitation of the study is the employment of a single item as an outcome measure, which may reduce the reliability of the responses, but we consider its correlation with a validated subjective well-being scale [29] to be satisfactory.

What can be done to increase resilience in medical students and ensure that future students are enabled to maintain stable, high levels of life satisfaction throughout medical school? The results of this study stress the importance of making time for personal and social life while in medical school, and the advantage of avoiding the use of passive, emotion focused coping, such as wishful thinking.

Findings from another study, which revealed that many medical students felt guilty for spending time on social activities and personal well-being even though they recognised the importance of doing so [13], may indicate that a change of social norms in medical school is necessary. Medical school educators could play an important role in such a change, by being good role models. In addition, reducing peer pressure is probably also an efficient means for reaching this goal. Evaluation of such interventions may be an important area for future research.

A review article on stress management in medical education found that participation in such programs gave promising results, but concluded that the studies had many limitations and that further research on stress reducing intervention in medical school is needed [41].

## Conclusion

In conclusion, this study shows that life satisfaction decreased somewhat during medical school. The medical students were initially as satisfied as other students, but the level of life satisfaction in their final year was lower than that of other comparable students. Medical students who sustained high levels of life satisfaction perceived medical school as interfering less with their social and personal life, and made less use of passive, emotion focused coping, such as wishful thinking, than did their peers. Medical schools should encourage students to try to achieve a balance between schoolwork and their social and personal lives, and emphasise the importance of healthy coping strategies, for instance, by providing stress management courses.

## Competing interests

The author(s) declare that they have no competing interests.

## Authors' contributions

KK acted as the principal investigator, RT was involved in designing the study, analysing the data and writing the paper, AF was involved in analyzing the data and writing the paper, EH was involved in designing the study and writing the paper, TG contributed to the interpretation of data and drafting of the manuscript, NTG was involved in initiating and designing the study and was responsible for collection of the data, PV and OE initiated and designed the study and supervised the collection of data, and were involved in writing the paper. All authors have read and approved the final manuscript.

## Pre-publication history

The pre-publication history for this paper can be accessed here:



## Figures and Tables

**Table 1:** Description of the explanatory variables in the longitudinal sample

Variable	Year measured	Range	Mean (*SD*) or percentages
Age (years)	First	19–39	21.6 (2.5)
Gender (female)	First		60%
BCI – vulnerability	First	0–9	3.7 (2.2)
BCI – intensity	First	0–9	5.2 (2.2)
BCI – control	First	0–8	3.2 (2.1)
PMSS – academic worries	Middle and final (sum)	6–28	15.7 (4.4)
PMSS – social and personal renunciations	Middle and final (sum)	6–24	14.5 (3.8)
WCCL – problem focused	Middle	11–38	23.3 (4.9)
WCCL – social support	Middle	1–20	12.2 (3.4)
WCCL – wishful thinking	Middle	3–38	18.5 (5.9)

Note: BCI = Basic Character Inventory; PMSS = Perceived Medical School Stress; WCCL = Ways of Coping Check List

**Table 2:** Levels of life satisfaction in medical school among students in the two clusters

Time of assessment in medical school	Students with stable high life satisfaction (n = 149)		Students with fluctuating life satisfaction (n = 87)	
	
	*Mean*	*SD*	*Mean*	*SD*
First year	5.9	0.9	5.2	1.0
Middle year	5.8	0.7	4.4	1.0
Final year	5.9	0.7	4.1	1.0

**Table 3:** Predictors of stable high life satisfaction in medical school

Variable	*OR*	95% CI	*p*-value
Age (years)			
*Unadjusted*	1.00	0.90–1.11	.96
***Adjusted***	**0.96**	**0.83–1.11**	**.62**
Gender (female)			
*Unadjusted*	0.86	0.50–1.48	.58
***Adjusted***	**1.30**	**0.59–2.88**	.52
BCI – vulnerability			
*Unadjusted*	0.80	0.71–0.91	< .01**
***Adjusted***	**0.88**	**0.74–1.06**	**.17**
BCI – intensity			
*Unadjusted*	1.10	0.97–1.24	.13
***Adjusted***	**0.99**	**0.85–1.17**	**.95**
BCI – control			
*Unadjusted*	0.95	0.84–1.08	.41
***Adjusted***	**1.18**	**0.99–1.40**	**.07**
PMSS – academic worries			
*Unadjusted*	0.80	0.74–0.87	< .01**
***Adjusted***	**0.92**	**0.83–1.01**	**.09**
PMSS – social and personal renunciations			
*Unadjusted*	0.77	0.70–0.84	< .01**
***Adjusted***	**0.76**	**0.68–0.85**	< .01**
WCCL – problem focused			
*Unadjusted*	1.07	1.01–1.14	.03*
***Adjusted***	**1.06**	**0.98–1.15**	**.14**
WCCL -social support			
*Unadjusted*	1.11	1.02–1.20	.02*
***Adjusted***	**1.07**	**0.96–1.18**	**.24**
WCCL – wishful thinking			
*Unadjusted*	0.90	0.85–0.95	< .01**
***Adjusted***	**0.93**	**0.87–1.0**	.04*

*Note*: Stable high life satisfaction is the reference group. OR = odds ratio; CI = confidence interval. * *p *< .05; ** *p *< .01. BCI = Basic Character Inventory; PMSS = Perceived Medical School Stress; WCCL = Ways of Coping Check List.
